# National Sanitary Organisation

**Published:** 1902-04-19

**Authors:** 


Vol. XXXII.—No. 812.] Edited by Sir Henry Burdett, K.C.B., and Solomon C. Smith, M.D., M.R.C.P. [April 19, 1902.

### National Sanitary Organisation.

If the government of national affairs were guided by anything like a correct appreciation of their relative importance, and if politicians were once to realise that, when they attain to power, the correction of grave legislative or administrative abuses within their respective departments becomes a matter of duty in relation to their fellow creatures, and a matter of honour in relation to the emoluments which they receive and the patronage which they enjoy, there would be some reason to hope that the recent and still continuing prevalence of small-pox would be used as a lever by which to bring about many important changes in the general sanitary administration of the country. A strong ministry, supported by the sudden wave of comparative enlightenment on questions of public health for which we are indebted to the somewhat exaggerated alarm which the so-called epidemic has occasioned, would be in a position to carry through many reforms which would materially contribute to the comfort and the healthiness of the general population, and would wipe away the stain of arrangements made when the care of public sanitary interests was first committed to a department of the Local Government Board, not only subordinate in its essential character, but also hampered at every step by the faulty principles on which it was constructed, and of which the present condition was foreshadowed in the scathing criticism with which Sir John Simon hailed its origin and its institution.

The then recent legislation in regard to public health had scared the Government of Mr. Gladstone, which was supported, as it naturally would be, by a vast number of small owners of insanitary property, whose suffrages were likely to be transferred to any other set of office seekers who would promise them immunity from control; and it was well understood that the chief function of Mr. Stansfeld, as the first president of the new Board, was to act in such a manner as to allay any alarm which persons of this description might at first not unnaturally experience. The action and its results might almost seem to justify the words written by Francis Jeffrey a hundred years ago, to the effect that statesmen and politicians, coming necessarily into contact with great vices and great sufferings, must gradually lose some of their horror for the first and much of their compassion for the last. Constantly engaged in contention, they cease pretty generally to regard any human beings as objects of sympathy or disinterested attachment; and mixing much with the most corrupt part of mankind, naturally come to regard the species itself with indifference, if not with contempt. Much the same sentiment was expressed a few years later by another keen observer, Balzac, in the words, "*Il n'y a plus de noblesse aujourd'hui, il n'y a plus qu'une aristocratie*;" and one consequence is that the feeling of duty, of obligation, in respect to public affairs, has to a large extent given way to a base contentment with the prospect of completing a Parliamentary session without defeat. The question of what is needed, or of what is right, is subordinated to the question of what is easy.

As long as any spirit of this kind is allowed to animate and control the proceedings of public men, it is of course hopeless to expect that questions of public sanitation will receive the consideration due to their importance, or to their actual bearing upon the prosperity of the empire. If they should ever come to do so, it is evident that the whole position of the central government in its relation to local authorities would require modification, especially in the direction of protecting neighbourhoods which are well administered against the evils liable to be inflicted upon them by the mal-administration of others in their vicinity. Few of the annual reports of the medical department of the Local Government Board have failed to contain evidence of the grossest neglect, in some place or other, of the most elementary sanitary requirements; of such malfeasance, for example, as the supply of excrement-polluted water to all the ratepayers of a district; and the press, to some extent, has been accustomed to republish more or less of such reports, and thus to call attention to the offences and to the offenders. In accordance with the immortal precepts of Dogberry, the local authority concerned has been bidden to stand; and, when it would not stand, the Central Board has been thankful to be rid of a knave. So far has the exposure been from producing reform that, in the majority of these cases, it forms part of the report that the very conditions described had been described before, five years, or ten years, or 15 years previously, and that in the interval nothing had been done. The need for central intervention arises, in such cases, from the fact that it is not the culprits alone who thus stew in their own juice. It is even possible that residents may acquire some degree of immunity against noxious influences by which they have long been surrounded; and that the most frequent victims may be travellers or passers through, or unfortunate persons who have temporary business in the places concerned. It would now be comparatively easy to compel the reform of conditions which thus constitute a public danger beyond the limits of their actual presence, and to organise the Central Board in such a manner as to render its intervention at once theoretically possible and practically operative. But we suppose that the "war," or the time required for answering the questions of Mr. Swift MacNeill, or Mr. Lloyd George, or the necessity of listening to Irish members while they exult in disasters to our soldiers, would be urged and accepted as sufficient reasons for neglect of everything which to plain men appears to occupy the position of a duty.

